# Deletion of the Gene for the Type I Interferon Inhibitor I329L from the Attenuated African Swine Fever Virus OURT88/3 Strain Reduces Protection Induced in Pigs

**DOI:** 10.3390/vaccines8020262

**Published:** 2020-05-30

**Authors:** Ana Luisa Reis, Lynnette C. Goatley, Tamara Jabbar, Elisabeth Lopez, Anusyah Rathakrishnan, Linda K. Dixon

**Affiliations:** 1 The Pirbright Institute, Ash Road, Pirbright, Surrey GU24 0NF, UK; lynnette.goatley@pirbright.ac.uk (L.C.G.); tamara.jabbar@pirbright.ac.uk (T.J.); anusyah.rathakrishnan@pirbright.ac.uk (A.R.); linda.dixon@pirbright.ac.uk (L.K.D.); 2 IRTA, Centre de Recerca en Sanitat Animal (IRTA-CReSA), Campus de la Universitat Autonoma de Barcelona, 08193 Bellaterra, Spain; elisabeth.lopezf@gmail.com

**Keywords:** African swine fever virus, interferon, I329L, vaccine

## Abstract

Live attenuated vaccines are considered to be the fastest route to the development of a safe and efficacious African swine fever (ASF) vaccine. Infection with the naturally attenuated OURT88/3 strain induces protection against challenge with virulent isolates from the same or closely related genotypes. However, adverse clinical signs following immunisation have been observed. Here, we attempted to increase the OURT88/3 safety profile by deleting I329L, a gene previously shown to inhibit the host innate immune response. The resulting virus, OURT88/3ΔI329L, was tested in vitro to evaluate the replication and expression of type I interferon (IFN) and in vivo by immunisation and lethal challenge experiments in pigs. No differences were observed regarding replication; however, increased amounts of both IFN-β and IFN-*α* were observed in macrophages infected with the deletion mutant virus. Unexpectedly, the deletion of I329L markedly reduced protection against challenge with the virulent OURT88/1 isolate. This was associated with a decrease in both antibody levels against VP72 and the number of IFN-γ-producing cells in the blood of non-protected animals. Furthermore, a significant increase in IL-10 levels in serum was observed in pigs immunised with OURT88/3ΔI329L following challenge. Interestingly, the deletion of the I329L gene failed to attenuate the virulent Georgia/2007 isolate.

## 1. Introduction

African swine fever (ASF) is a devastating disease of domestic pigs and wild boar, which can result in the death of almost all infected animals. ASF has spread in many countries in sub-Saharan Africa, Russian Federation, Europe and most recently to China and other S. E. Asian countries. Information on disease outbreaks is updated daily (OIE WAHIS https://www.oie.int/wahis_2/public/), and the situation in Asia is summarised weekly by the United Nations Food and Agriculture Organisation (FAO) (http://www.fao.org/ag/againfo/programmes/en/empres/ASF/situation_update.html).

African swine fever virus (ASFV) is a large cytoplasmic DNA virus and is the only member of the *Asfarviridae* family [1]. The genome of 170–193 kbp contains about 150–170 genes. These include many that are not essential for virus replication in cells but have roles that include the evasion of host defences [1]. Several inhibitors of type I interferon (IFN) responses have been identified, including members of the virus multigene families (MGF) 360 and 505/530 and the DP96R/UK protein. The deletion of multiple members of MGF 360 and 505 results in the attenuation of virulent isolates, including genotype I Benin 97/1, genotype II Georgia, and Pr4 [2,3,4]. The deletion of the DP96R/UK gene also resulted in the attenuation of the E70 isolate, although it did not reduce virulence of the genotype II Georgia isolate [5,6]. Previously, in cultured macrophages infected with virulent ASFV, the induction of type I IFN and activation of IFN responses was inhibited, whereas, in those infected with virulent ASFV, from which multiple copies of MGF360 or 505/530 were deleted, varying levels of IFN-*β* or interferon stimulated genes were expressed [2,4]. Increased type I IFN-*β* mRNA transcripts were also observed in macrophages infected with the naturally attenuated ASFV isolate OURT88/3 isolate [4]. This has a deletion of similar numbers of MGF360 and MGF505/530 genes as the ASFV gene deletion mutants BeninΔMGF and Pr4. Thus, there is a good correlation between the increased induction of type I IFN and the attenuation of ASFV.

The ASFV I329L protein is a predicted type I transmembrane protein that contains motifs typical of Toll-like receptors [7,8]. These include four leucine-rich repeats (LRRs) in the extracellular domain and a weak homology with the cytoplasmic Toll-interleukin-1 receptor (TIR) domain of Toll-like receptor 3 (TLR3). This domain mediates interactions between TLRs and cytoplasmic adaptor proteins. These similarities suggested that the I329L protein may act as a TLR antagonist by inhibiting the activation of signalling pathways downstream of TLR3 and possibly other TLRs. Transiently expressed I329L inhibited the activation of IFN-β promoter and NF-κB-dependent luciferase reporters following the activation of TLR3 by the double-stranded RNA mimic polyinosinic:polycytidylic acid (poly IC) or of the downstream pathway by overexpression of the TIR-domain-containing adapter-inducing interferon-β (TRIF) adaptor protein. Protein structure modelling suggested that I329L may function as a TLR3 decoy by formation of I329L-TLR3 heterodimers, thus inhibiting the downstream type I IFN induction pathway [9]. The transient expression of I329L inhibited the secretion of IFN-β into cell supernatants, confirming that the expression of the protein inhibits type I IFN induction [8].

Although exogenously expressed I329L protein has been shown to reduce type I IFN production from cells, its role during the virus infection of cells or pigs has not previously been investigated. In the current study, we deleted the I329L gene from the genome of the natural attenuated genotype I isolate OURT88/3 (OURT88/3ΔI329L) and from the genotype II virulent Georgia 2007/1 isolate (GeorgiaΔI329L). We hypothesized that the I329L deletion would result in increased amounts of type I IFN being secreted by infected cells, resulting in the inhibition of viral replication in vivo and, importantly, the promotion of the adaptive immune response.

The results show that this gene deletion did not have any significant effect on replication of the viruses in cells. However, porcine macrophages infected with OURT88/3ΔI329L expressed significant higher amounts of type I IFN than the ones infected with wild-type (wt) OURT88/3.

Pigs infected with the GeorgiaΔI329L virus developed high viremia as well as clinical and pathological signs typical of acute ASFV. The deletion of I329L from the OURT88/3 strain did not result in a reduction in clinical signs but unexpectedly reduced the level of protection against challenge. Thus, an effect of deleting I329L was observed when it was deleted in combination with other type I interferon inhibitors from an attenuated strain but not singly from a highly virulent strain.

## 2. Materials and Methods

### 2.1. Viruses and Cells

The OURT88/3 low virulence non-haemabsorbing genotype I ASFV isolate and the virulent Georgia 2007/1 genotype II isolate have been described previously [10,11]. These viruses were grown in primary pig macrophage cultures from bone marrow (PBMs). Virus titres were determined by end-point dilution in PBM cultures. The virus was detected by immunofluorescence using a monoclonal antibody against virus protein p30/CP204L (mouse monoclonal IgG1 antibody clone C18, Pirbright, UK) and an appropriate secondary antibody. Virus titres were calculated as the amount of virus infecting 50% of the PBM cultures (TCID_50_/mL).

### 2.2. Recombinant Viruses

Right and left genome fragments of approximately 700 to 800 bp flanking the I329L gene were amplified by PCR. The following primers were used for the left flanking fragment encompassing genome positions 157245 to 158064: CATATGTTTTTGAAGCGTTCTAAAAAACATC and ATGTGTGGTTTATTTTAGTATG. The right flanking fragment was amplified with the GAGTTCTTTACCAAAGCC and GGAGGATGACACATATATCTTAACC primers comprising genome positions 158716 to 159434 of the OURT88/3 isolate. Similar fragments were amplified from the Georgia 2007/1 isolate. The obtained DNA fragments were cloned into the pLoxPVP72GUSLoxP vector to construct the pΔI329LGUS plasmids. Pig alveolar macrophages (PAMs) were infected with OURT88/3 or Georgia 2007/1 isolates and transfected with the pΔI329LGUS plasmids using TransIT-LT1 (Mirus Bio, Madison, WI, USA). Recombinant viruses expressing the *β*-GUS gene were identified by incubation with 5-bromo-4-chloro-1H-indol-3-yl *β*-D-glucopyranosiduronic acid and purified by limiting dilution. Viral genomic DNA was purified from supernatants from infected porcine macrophages using MagVet™ Universal Isolation Kit (Life Technologies). The analysis of viral DNA was carried out by PCR amplification using primers binding within the I329L deletion (GGACTGTTTGCTGAGGTTGTATG and CCCTTATACTACTTCCTACTGAAACAGG) or flanking regions (GGTTCTATAAATAGCATACTGTACAG and CTGCTGGCATTTCATGCACTTG).

### 2.3. Quantification of IFN-β Transcripts

The expression of IFN-*β* was quantified as described previously [4]. Briefly, PAMs (5 × 10^5^ cells) were infected with ASFV or mock infected. At selected times, RNA was extracted (RNeasy mini kit, Qiagen, Hilden, Germany) and equal amounts were used as a template to synthesise cDNA (Superscript III reverse transcriptase kit, Invitrogen, San Diego, CA, USA). IFN-*β* transcripts were measured by quantitative real time PCR (qPCR) using a power SYBR Green Master Mix (Thermo Fisher Scientific, Hemel Hempstead, UK). IFN-*β* and Glyceraldehyde 3-phosphate dehydrogenase (GAPDH) copy numbers were calculated by the standard curve method, and the results are presented as the IFN-*β*/GAPDH ratio. Assays were carried out in duplicate.

### 2.4. Quantification of IFN-α in Supernatants and Pig Sera

PBMs were purified from bone marrow suspensions by gradient centrifugation and seeded at 1 × 10^6^ cells per well (24 well plate). Cells were infected with the different ASFV isolates at a multiplicity of infection (MOI) of 1 (1.45 × 10^6^ TCID_50_ per well). After 1 h of incubation at 37 °C, the inoculum was removed and fresh medium was added. The infected cells were further incubated for 16 h and the supernatants were collected. The amount of IFN-*α* in supernatants or pig sera was quantified by an in-house ELISA. Briefly, Maxisorp plates (Nunc, Roskilde, Denmark) were coated overnight at room temperature with anti-pig IFN-*α* antibody (clone K9) at 0.5 µg/mL in 0.5 M carbonate-bicarbonate coating buffer. Plates were washed with wash buffer (0.05% Tween 20 in PBS) and blocked with 1% BSA in PBS. Samples and standards were then added and the plates were incubated at room temperature for 2 h. After washing, detection antibody (biotinylated anti-pig IFN-*α* antibody—clone F17) diluted 1:5000 in blocking buffer was added and the plates were incubated at room temperature for 2 h. The plates were then washed, incubated with Streptavidin horseradish peroxidase (HRP) and finally developed with 3,3′,5,5′-Tetramethylbenzidine (TMB) substrate (R and D Systems, DY999). After stopping the reaction with 2 N H_2_SO_4_, the absorbances were read at 450 nm.

### 2.5. Virus Growth Analysis

PBMs were infected at an MOI of 0.3 with the OURT88/3, OURT88/3ΔI329L, Georgia 2007/1 or GeorgiaΔI329L. Cells and supernatants were collected at different times post-infection and subjected to 3 freeze-thaw cycles. Cellular debris was removed by centrifugation, and virus titres were determined as above.

### 2.6. Pig Immunisation and Challenge

The experiments were conducted in the SAPO4 high containment large animal isolation units at the Pirbright Institute under Home Office License PPL70/8852. One group of 5 and one group of 6 Large white/Landrace pigs of 15–20 kg weight, in separate rooms, were immunised by the intramuscular route with 10^4^ TCID_50_ in 1 mL of the wild-type OURT88/3 virus (OURT88/3_wt 1–5) or the OURT88/3 virus with the I329L gene deleted (OURT88/3_del 1–6). After 21 days, all pigs were challenged by the intramuscular route with 10^4^ TCID_50_ of virulent strain OURT88/1, in parallel with 3 control non-immune pigs in a separate room. Pigs were observed and scored for the development of clinical signs, including fever, loss of appetite, lethargy and external signs of haemorrhage [12]. At defined humane endpoints, pigs were euthanized by an overdose of barbiturates. Those pigs that survived challenge were terminated at the end of the experiment 21 days post-challenge. Macroscopic lesions were scored at post-mortem examination [13]. In a second experiment, a group of 6 pigs were infected with 10^4^ TCID_50_ of the GeorgiaΔI329L virus by the intramuscular route and clinical signs were scored as above. In both experiments, blood and serum samples were collected at defined time points during the experiment and tissues at post-mortem. These samples were stored at −80 °C.

### 2.7. Measurement of Virus Genome Copy Numbers by Quantitative PCR

Virus genome copy numbers in whole blood were measured by quantitative PCR (qPCR) [12,14] and expressed as genome copies per ml of blood.

### 2.8. IFN-γ ELiSpot Assays

Peripheral blood mononuclear cells (PBMC) were purified from EDTA blood using gradient centrifugation. ELIspot plates (MAIPS4510, Millipore, Burlington, MA, USA) were coated overnight at 4 °C with 4 µg/mL anti-porcine IFN-*γ* mAb P2F6 in 0.5 M carbonate-bicarbonate coating buffer and then washed with PBS. Cells were plated in duplicate at two different dilutions, typically 8 × 10^5^ and 4 × 10^5^ per well in Roswell Park Memorial Institute (RMPI) supplemented with 10% foetal calf serum, 1 mM sodium pyruvate, 50 µM 2-mercaptoethanol, 100 IU/mL penicillin and 100 µg/mL streptomycin. Cells were then incubated overnight in a final volume of 200 µL with 10^5^ haemadsorption (HAD) units of OURT88/1 or an equivalent volume of mock inoculum, or 2.5 µg/mL phytohaemagglutinin as a positive control. Cells were lysed by incubating for 5 min in water and then washed with PBS. Biotinylated anti-porcine IFN-*γ* mAb P2C11, followed by streptavidin conjugated to alkaline phosphatase was used to visualise spots that were then counted using an ELIspot Reader System (AID). The number of spots per well was converted into the number of spots per million cells and the mean of duplicate wells plotted.

### 2.9. ELISA Assays

The presence in serum of antibodies against ASFV protein VP72/B646L were measured using a commercial competitive ELISA (INGEZIM PPA Compac, Ingenasa, Madrid, Spain). IL-10 was detected in serum using a commercial kit (R and D systems). IFN-*α* in serum was quantified as described in Section 2.4.

### 2.10. Statistical Analysis

Statistical analysis was performed using GraphPad Prism7 software (GraphPad Software Inc., San Diego, CA, USA). Differences between groups were determined using unpaired *t*-test or two-way ANOVA followed by Tukey’s multiple comparison test. The log-rank test (Mantel–Cox) was used to compare survival after challenge.

## 3. Results

### 3.1. Deletion of the I329L Gene Does Not Reduce Replication of the OURT88/3 and Georgia 2007/1 Strains in Macrophages

**Figure 1:**
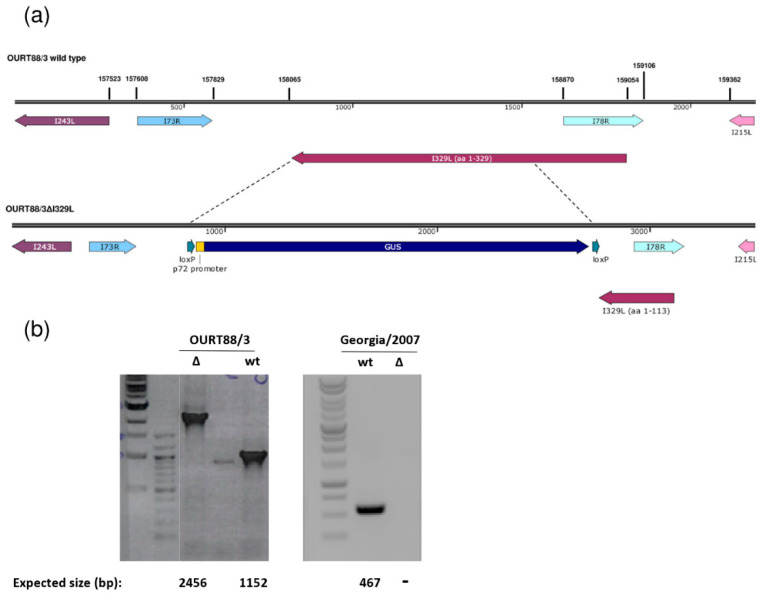
Deletion of the I329L gene from the OURT88/3 and Georgia 2007/1 genomes. (**a**) The position and direction of the I329L gene and neighbouring genes on the wild-type OURT88/3 genome are shown on the top panel. Arrows indicate the direction in which the genes are read. The bottom panel shows details of the construction of the I329L gene deletion. The *β*-Glucoronidase (GUS) gene under control of the African swine fever virus (ASFV) B646L gene promoter and flanked by loxP sites was used to replace the I329L gene, excluding the portion encoding residues 1 to 113. This fragment was retained to avoid disrupting the overlapping I78R gene, which is read from the opposite strand. (**b**) Analysis of genomic viral DNA by PCR. Viral DNA was extracted from wild-type (wt) and delta I329L (Δ) viruses. Specific fragments were amplified by PCR and the products were analysed by agarose gel electrophoresis. For OURT88/3, the fragments encompassing the flanking regions of the I329L deletion are shown and the expected fragment sizes are indicated below each lane. For Georgia 2007/1, a fragment within the deletion was obtained for the wt but not for the deletion mutant virus as expected.

The I329L gene was deleted from the genome of the attenuated ASFV OURT88/3 or the virulent Georgia 2007/1 isolate and replaced with the *β*-glucoronidase gene as described in the Materials and Methods. Since the I329L and the I78R open reading frames (ORFs) overlap, the deletion was designed to preserve I78R expression, by retaining the I78R coding region and 166 base pairs upstream of its start codon. This results in the truncation of I329L, with the residues 1 to 113 remaining (Figure 1). Therefore, the signal peptide and the N-terminal region of the type I transmembrane I329L protein that is predicted to be highly glycosylated have the potential to be expressed by the deletion mutant virus. All the other predicted domains were removed (leucine-rich repeats, transmembrane domain and cytoplasmic domain). Since it would lack the transmembrane domain required for membrane anchor and the cytoplasmic domain, which is predicted to impact on TRIF activity, we expect that the protein is no longer functional.

**Figure 2:**
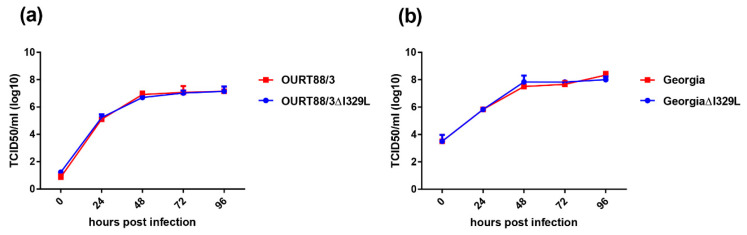
Deletion of the I329L gene does not reduce the replication of the OURT88/3 (**a**) and Georgia 2007/1 (**b**) strains in primary porcine bone marrow cells. Virus titres in TCID50/mL on the y axis from cultures of porcine bone marrow cells infected for times between 0 and 96 h (x axis) with wild-type (red) or ΔI329L (blue) viruses at a low multiplicity of infection.

The replication kinetics of the OURT88/3ΔI329L and GeorgiaΔI329L viruses in bone marrow derived porcine macrophages was compared with that of parental viruses over a 96 h period using a low multiplicity of infection. The results (Figure 2) show no significant difference in the replication kinetics of the viruses. For OURT88/3, both viruses reached a plateau of between 10^6.6^ and 10^7^ TCID_50_/mL by 48 h, which was maintained for the rest of the time course. For the Georgia viruses, a plateau was also reached at 48 h with values between 10^7.5^ and 10^8.1^ TCID_50_/mL. Thus, the deletion of the I329L gene did not reduce virus replication in macrophages.

### 3.2. Deletion of the I329L Gene from the OURT88/3 Isolate Results in Increased Type I IFN Production

**Figure 3:**
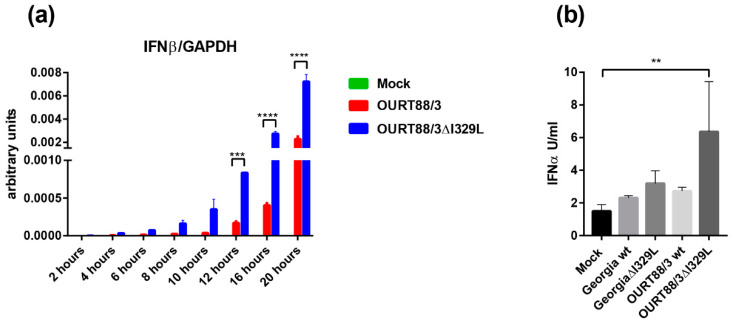
Expression of type I interferon (IFN) in macrophages infected with wild-type and I329L deletion mutants. Panel (**a**) shows IFN-*β* transcripts relative to GAPDH. Pig alveolar macrophages (PAMs) were mock infected or infected with wild-type OURT88/3 or OURT88/3Δ329L viruses at an multiplicity of infection (MOI) of 1. At different times post-infection (x-axis), RNA was extracted and copy numbers for IFN-*β* and GADPH were quantified by quantitative real time PCR (qPCR) (y-axis). Panel (**b**) shows IFN-*α* detected in supernatants from porcine bone marrow cell cultures infected with wild-type or I329L gene-deleted ASFV isolates. PBM cells were mock infected or infected with wild-type Georgia or OURT88/3 isolates and I329L gene deletions from these viruses as indicated on the x-axis at an MOI of 1. At 16 h, post-infection levels of IFN-*α* were assayed and are shown as units per/mL on the y axis. The results shown are averages from infections of cells from 3 different outbred pigs. Asterisks indicate statistically significant differences between the indicated samples (** *p* ≤ 0.01; *** *p* ≤ 0.001; **** *p* ≤ 0.0001).

PAMs were infected at an MOI of 1 with OURT88/3, OURT88/3ΔI329L or mock infected. At different times post-infection, levels of IFN-*β* and GAPDH transcripts were quantified by qPCR. IFN-*β* mRNA was detected at very low levels in mock infected cells throughout the time course. In contrast, in cells infected with ASFV, the levels of IFN-*β* steadily increased from 4 h post-infection. Importantly, cells infected with OURT88/3ΔI329L expressed significantly higher levels of IFN-*β* than cells infected with the parental virus at 12, 16 and 20 h post-infection. (Figure 3a).

PBMs were infected at an MOI of 1 with OURT88/3, OURT88/3ΔI329L, Georgia 2007/1 and GeorgiaΔI329L isolates or mock-infected. Supernatants were collected at 16 h pi and levels of IFN-*α* were measured by ELISA. Figure 3b shows the results obtained using cells from three outbred pigs. The levels of secreted IFN-*α* were very low and almost indistinguishable from mock-infected cells except for the ones infected with OURT88/3ΔI329L (*p* = 0.0064). These results confirm a role for I329L in the modulation of the host IFN response in the context of ASFV infection.

### 3.3. Immunisation and Challenge of Pigs

#### 3.3.1. Clinical and Post-Mortem Signs

An immunisation and challenge experiment was carried out to determine if deletion of the I329L gene from the OURT88/3 genome had altered clinical signs, the levels of virus replication, immune responses and protection. A group of six pigs was immunised with the OURT88/3ΔI329L (del_1 to 6) virus and five pigs with the parental OURT88/3 (wt_1 to 5) virus by the intramuscular route using 10^4^ TCID_50_. At 21 days post-immunisation, all pigs in these groups were challenged with 10^4^ virulent OURT88/1 virus, in parallel with three non-immune control pigs present in a different room.

**Figure 4:**
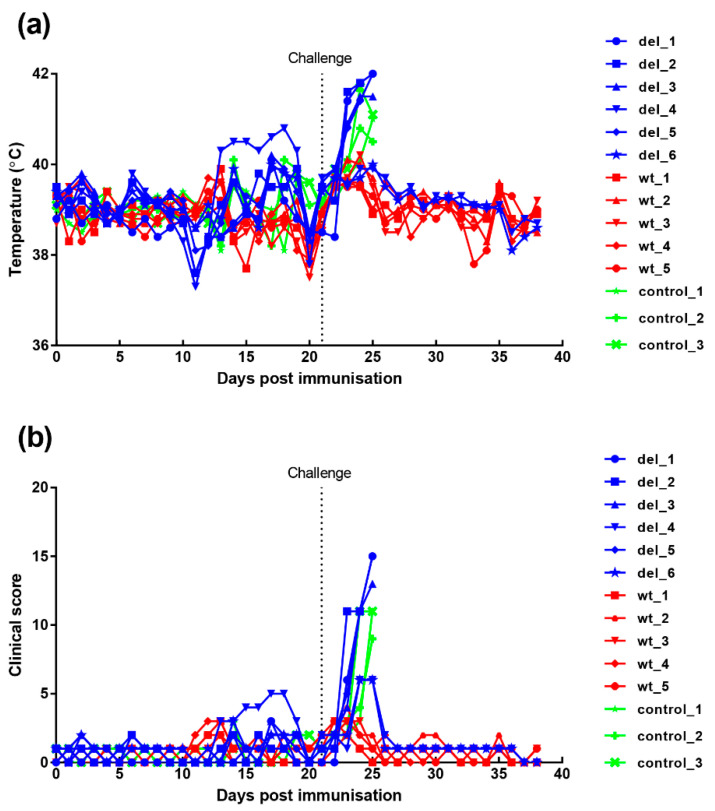
Clinical scores and temperatures of pigs following immunisation with OURT88/3 or OURT88/3ΔI329L and challenge with virulent virus. Panel (**a**) shows temperatures and panel (**b**) scores all clinical signs on the y-axis. Days post-immunisation are shown on the x-axis. The blue symbols and signs show those pigs immunised with the OURT88/3ΔI329L virus (del_1–6) and red with OURT88/3 (wt_1–5). Information on the control pigs is shown in green (control_1–3).

Clinical signs post-immunisation and challenge (Figure 4) show that, after immunisation, one pig, (del_4) from the group immunised with the OURT88/3Δ329L deletion virus, developed a temperature of between 40.3 and 40.8 °C between days 13 and 19. This coincided with the appearance of a swelling at the vaccination site. After anti-inflammatory and antibiotic treatment, the swelling reduced and temperature decreased. Pig del_6 had a reduced appetite on 2 days. Apart from this, no further signs were observed in this group before challenge.

**Figure 5:**
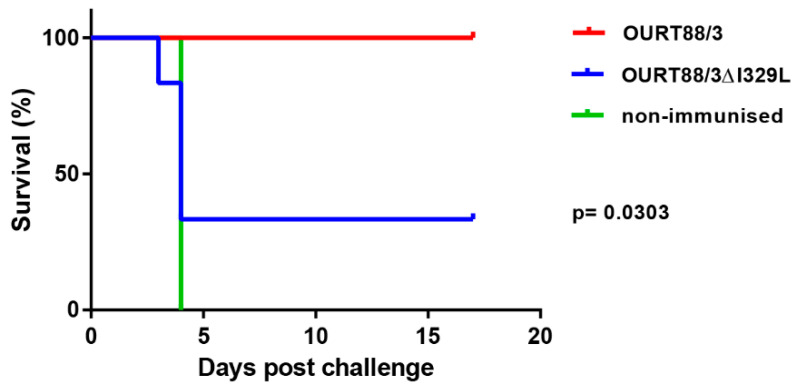
Survival of pigs immunised with OURT88/3 or OURT88/3ΔI329L or control pigs after challenge with virulent virus. The percentage of pigs that survive challenge is shown on the y-axis and days post-challenge on the x-axis. Pigs immunised with OURT88/3 are shown in red, with OURT88/3ΔI329L in blue. Control pigs are shown in green.

After challenge, four pigs (del_1, 2, 3, 5) from the group immunised with OURT88/3Δ329L virus developed signs of acute ASFV and were terminated when they reached a defined humane endpoint at day 3 (del_ 2) or 4 (del_1, 3, 5) post-challenge. The 3 non-immune control pigs were terminated at day 4 post-challenge. In contrast, all pigs immunised with the OURT88/3 parental strain survived challenge showing no clinical signs. Thus, the deletion of the I329L gene reduced protection (*p* = 0.0303) from 100% in pigs immunised with OURT88/3 parental virus to just two out of six in pigs immunised with OURT88/3Δ329L (Figure 5). A post-mortem examination of pigs terminated before the end of the experiment, when they reached the defined humane endpoint, showed gross lesions typical of acute ASFV, including enlarged and haemorrhagic spleen and lymph nodes. Pig del_5 also showed cyanosis on the ear tips. The pigs that survived the challenge showed no post-mortem signs apart from slight haemorrhage in the renal lymph node in pig del_4.

#### 3.3.2. Virus Genome Copy Numbers in Blood

**Figure 6:**
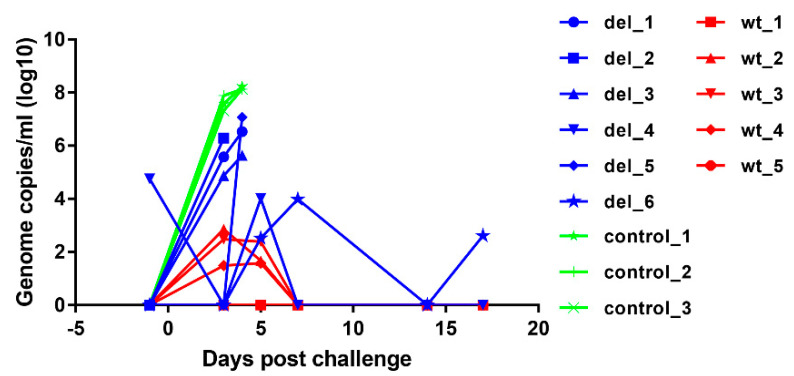
ASFV genome copies detected in blood from pigs immunised with OURT88/3 or OURT88/3ΔI329L or control pigs at different times post-challenge with virulent virus. ASFV genome copies per ml are shown on the y-axis and days post-challenge on the x-axis. The results for pigs immunised with OURT88/3 are shown in red (wt_1–5), with OURT88/3ΔI329L in blue (del_1–6) and control pigs in green (control_1–3).

To assess the levels of virus replication in pigs after immunisation and challenge, virus genome copy numbers per ml of blood were measured by qPCR (Figure 6). After immunisation, no virus was detected in blood except in one pig, del_4, which had 10^4.75^ ASFV genome copies per ml of blood on the day before challenge but this reduced to an undetectable level by 3 days after challenge. At day 5 post-challenge, this pig had 10^4^ genome copies per ml of blood, but no further viremia and no clinical signs were detected and the pig survived until the termination of the experiment. None of the other pigs had detectable virus on the day of challenge. The other pig that survived, del_6, had intermittent viremia of 10^2.6^ to 10^4^ genome copies per ml after challenge. Three of the pigs that were immunised with OURT88/3ΔI329L and were not protected had genome copy numbers of 10^4.9^ to 10^6.3^ per mL by day 3 post-challenge, and the pig with highest genome copy numbers was euthanised on that day (del_2). The three other non-protected pigs (del_1, 3, 5) had viremia of 10^5.6^ to 10^7^ genome copies per ml of blood at the time of euthanasia on day 4. In contrast, pigs in the group that were immunised with parental OURT88/3 virus had either no detectable viremia (pigs wt_1 and wt_5) or transient low levels between 10^1.5^ and 10^2.9^ per ml of blood on days 3 and 5 post-challenge. As expected, the control non-immune pigs developed high levels of viremia (10^7.3^ to 10^7.9^) by day 3 post-challenge rising to above 10^8^ on day 4 when they were euthanised. The comparison of genome copies per mL showed a significantly lower level in pigs immunised with the OURT88/3ΔI329L or OURT88/3 compared to the control pigs (*p* value 0.0026 or 0.0002, respectively) at day 3 post-challenge. No significant difference was observed between animals immunised with OURT88/3 and OURT88/3ΔI329L.

### 3.4. Immune Responses to Infection

**Figure 7:**
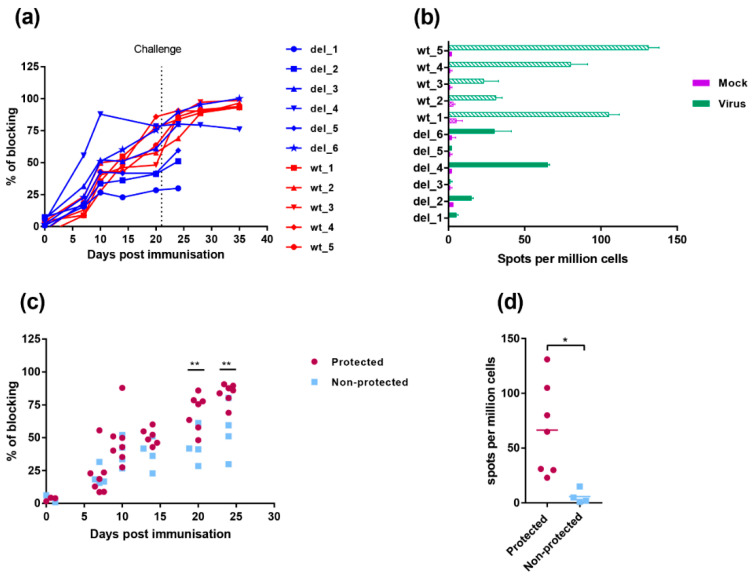
Immune responses in pigs immunised with OURT88/3 and OURT88/3ΔI329L. Panel (**a**) shows anti-ASFV antibodies detected by a blocking ELISA at different days post-immunisation. The y-axis shows the percentage of blocking, and the x-axis shows days post-immunisation. The results for pigs immunised with OURT88/3 are shown in red (wt_1–5), with OURT88/3ΔI329L in blue (del_1–6). Panel (**c**) compares antibody response in pigs that survived challenge (protected, magenta) with those that did not survive (non-protected blue). Panel (**b**) shows the numbers of IFN-γ-producing cells detected by ELIspot assay following the stimulation of lymphocytes with ASFV. The x-axis shows IFN-γ-producing cells (spots) per million of cells detected, and the y-axis shows the source of samples. The results from mock-treated (purple) and ASFV-stimulated lymphocytes (green) are shown. Panel (**d**) compares numbers of IFN-γ-producing cells detected in protected versus non-protected pigs. Asterisks indicate statistically significant differences between groups (* *p* ≤ 0.05; ** *p* ≤ 0.01).

The antibody responses to infection were measured using a competitive ELISA test against the major capsid protein VP72/B646L (Figure 7a). The antibody response to VP72/B646L was first detected weakly by day 7 and continued to rise in most pigs during the experiment. One pig, del_4, in the group immunised with OURT88/3ΔI329L had a higher antibody response on days 7 and 10 post-immunisation than other pigs in this group or the group immunised with OURT88/3. However, no significant difference was detected between responses when all pigs in the different groups were combined. Antibody responses in the pigs protected against challenge compared to non-protected pigs (Figure 7c) showed a significantly lower response in the non-protected pigs at days 20- and 24-days post-immunisation (−1 and +3 post-challenge) (*p* value 0.0090 and 0.0039, respectively).

Cellular responses were measured by the numbers of cells producing interferon gamma using an ELIspot assay, following stimulation with ASFV of PBMC collected on the day before challenge. Figure 7b shows that individual pigs from the group immunised with OURT88/3ΔI329L had low or undetectable responses. Pig del_4 showed the highest response in this group, higher than two of the pigs from the group immunised with OURT88/3. Pig del_6 had a response similar to the two lowest responders from the wt group (wt_2 and wt_3) and other pigs from the group OURT88/3ΔI329L showed no or very low responses. Thus, the two pigs that were protected from this group (del_4 and del_6) showed the highest response to ASFV in this assay. PBMCs from pigs immunised with OURT88/3 all responded to ASFV stimulation and pigs wt_1, wt_4 and wt_5 showing highest responses. The responses in the pigs that were protected were significantly higher (*p* value 0.0193) compared to those in non-protected pigs (Figure 7d).

#### 3.4.1. IFN-α in Serum

**Figure 8:**
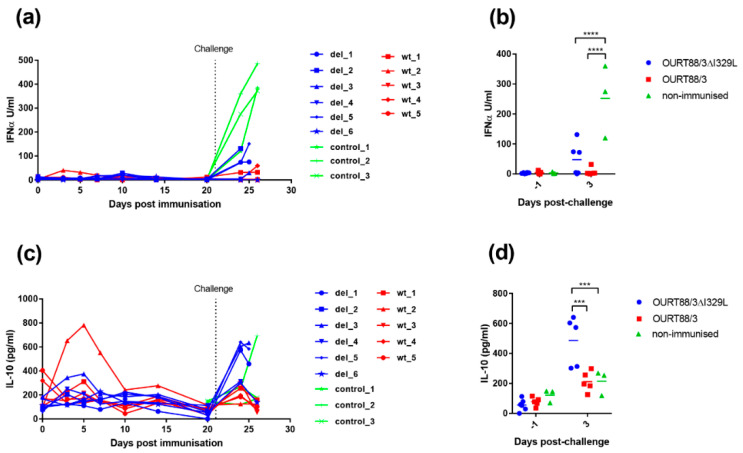
Detection of IL-10 and IFN-*α* in sera from pigs after immunisation and challenge. Panel (**a**) shows IFN-*α* and panel (**c**) IL-10 detected in serum at different days post-immunisation. The y-axis shows the amount detected in Units per ml, and the x-axis shows the days post-immunisation. The results for pigs immunised with OURT88/3 are shown in red (wt_1–5), with OURT88/3ΔI329L in blue (del_1–6) and control pigs in green (control_1–3). Panels (**b**,**d**) show the results post-challenge analysed to determine statistical differences between groups. Asterisks indicate statistically significant differences between groups (*** *p* ≤ 0.001; **** *p* ≤ 0.0001).

We showed that I329L deletion increases type I IFN production in vitro (Figure 3). To further explore the role of I329L in the modulation of the host IFN response, the levels of IFN-*α* were measured in the serum of vaccinated and control pigs. As shown in Figure 8a,b, the levels of IFN-*α* were very low before challenge, except for pig wt_2. This pig showed a slight increase in IFN-*α* on days 3 and 5 post-immunisation that returned to baseline levels at day 10. After challenge, a significant increase in IFN-*α* was observed in sera from control non-vaccinated pigs compared to the pigs immunised with the wild-type or the deletion mutant OURT88/3 viruses (*p* < 0.0001).

#### 3.4.2. IL-10 in Serum

The IL-10 cytokine response was measured in serum to determine if there was a correlation between levels of this anti-inflammatory cytokine and the lack of protection in pigs. As shown in Figure 8c,d, the levels of IL-10 were low before challenge except in one pig, wt_2. This pig had a peak of IL-10 between days 3 and 7 post-immunisation but levels then declined to that of other pigs. After challenge, the levels of IL-10 increased sharply in serum from 3 of the non-protected pigs del_1, 3 and 5 and in one of the control pigs. The levels of IL-10 in serum from all the other pigs increased to levels that were less than half of that in pigs del_1, 3 and 5. The levels of IL-10 were significantly higher at day 3 post-challenge in the group immunised with OURT88/3ΔI329L compared to those immunised with OURT88/3 or the control group (*p* = 0.0002 or 0.0008, respectively).

### 3.5. Infection of Pigs with An I329L Gene Deleted Georgia 2007/1 Strain

**Figure 9:**
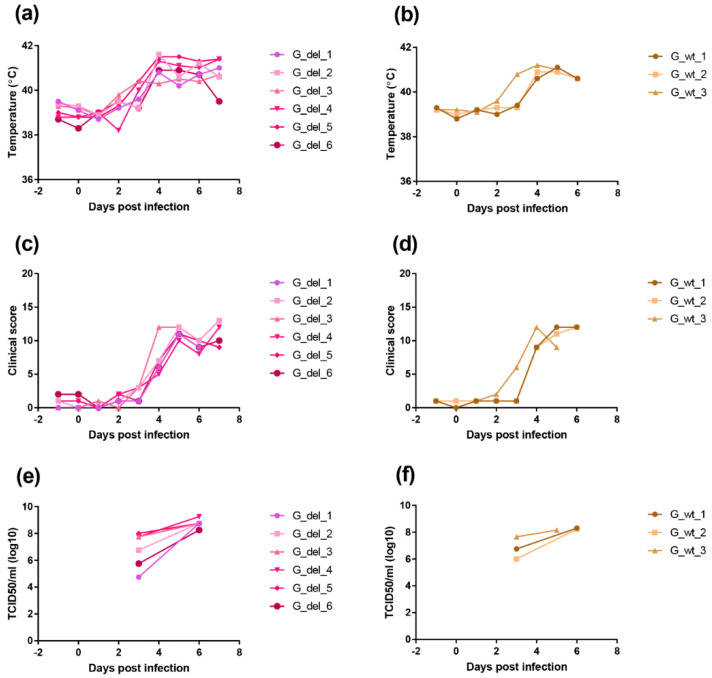
Clinical scores and temperatures of pigs following infection with GeorgiaΔI329L or Georgia 2007/1 wild type. The panels (**a**,**b**) show temperatures and panels (**c**,**d**) clinical scores following infection of pigs with GeorgiaΔI329L (G_del_1 to 6) or Georgia wild type (G_wt_1 to 3). The panels (**e**,**f**) show TCID_50_ per ml of blood (y-axis) at different days post-infection.

We deleted the I329L gene from the virulent genotype II ASFV Georgia 2007/1 isolate to determine the effect on virus replication and pathogenesis in pigs. A group of six pigs were infected with 10^4^ TCID_50_ of the GeorgiaΔI329L virus and a group of three pigs were infected with the Georgia 2007/1 virus by the intramuscular route. Clinical signs were scored and blood samples collected at different days post-infection (Figure 9).

In the group infected with GeorgiaΔI329L, three pigs showed an increased temperature above 40 °C by day 3 and all had an increase by day 4 rising above 41 °C in all except 1 pig. Other clinical signs, including lethargy and loss of appetite, were also observed. All pigs were euthanized on day 7 when they reached a predefined moderate severity end-point. At post-mortem, all pigs had macroscopic lesions typical of acute ASFV, including enlarged and darkened spleen, haemorrhagic lymph nodes and petechia on the kidneys.

A similar disease course was observed in the group infected with the wild-type virus, with one pig showing temperature above 40 °C by day 3 and the remaining pigs showing increased temperatures from day 4. Other clinical signs, such as lethargy and loss of appetite, were also present as well as lesions typical of acute ASFV at post-mortems.

Infectious virus numbers in blood were measured by virus titration. At day 3 post-infection, levels varied between 10^4.75^ and 10^8^ TCID_50_ per ml and by day 6 levels were between 10^8.25^ and 10^9.25^ TCID_50_ per ml for the pigs infected with GeorgiaΔI329L. Pigs infected with the parental virus showed viremias between 10^6^ and 10^7.65^ TCID_50_ per ml at day 3 post-infection and between 10^8.15^ and 10^8.3^ TCID_50_ per ml by day 5 or day 6 post-infection. The levels of viremia confirmed the results from clinical and post-mortem scoring (14), and indicate that deletion of the I329L gene does not attenuate the Georgia 2007/1 strain.

## 4. Discussion

ASFV codes for many inhibitors of innate immunity, including inhibitors of type I interferon induction. In general, little is known about the mechanisms by which these proteins act. Most, for example, the MGF360, MGF505 and DP96R genes have no similarities with other known proteins, although some defined motifs are present. One exception is the type I transmembrane I329L protein, which shares similarity with, and is proposed to act as an antagonist of, TLR3, thus inhibiting the downstream pathway of IFN induction. The deletion of multiple copies of MGF360 and 505 or of DP96R genes can result in attenuation of virulent ASFV, with some variation in the level of attenuation between isolates. In most cases, pigs immunised with attenuated viruses, from which genes coding for inhibitors of type I IFN have been deleted, are protected against challenge with parental virulent virus. This is presumed to result from the increased induction of interferon stimulated genes which induce innate and adaptive immune responses.

In our current study, we investigated the effect of deleting the I329L gene from an already attenuated isolate, OURT88/3, and from a highly virulent isolate. Our results confirm that the gene could be deleted without affecting the levels of virus replication in porcine macrophage cultures. Importantly, the deletion mutant OURT88/3ΔI329L induced higher levels of IFN-*β* transcripts, in infected porcine primary macrophages, than the wild-type virus. Furthermore, we observed a small but significant increase in IFN-*α* levels in supernatants from OURT88/3ΔI329L macrophages compared to mock-infected cells. Remarkably, Razzuoli et al. showed that the infection of porcine macrophages with NHP/68 (an attenuated genotype I isolate, similar to OURT88/3) significantly increased the number of several IFN-*α* subtype transcripts [15]. However, in agreement with our results from the OURT88/3 wild type, they could not detect any significant increase in IFN-*α* in supernatants from infected cells. It is therefore possible that ASFV also interferes with post-translation modifications/secretion of IFN. It is also worth mentioning that type I IFN secretion initiates a positive feedback loop, priming neighbouring cells to produce more IFN. Hence, even low levels of IFN secreted by the first infected cells may have a significant impact at the virus replication sites. Taken together, our results suggest that I329L has evolved to modulate the host innate immune response and supports previous data from transiently transfected cells [7,8].

We anticipated that the deletion of I329L from the OURT88/3 isolate would further increase the expression of type I IFN and enhance the host immune response, thus preventing the occurrence of adverse clinical signs following immunisation. Our hypothesis was not confirmed, and, instead, we observed a decrease in protection in pigs immunised with OURT88/3ΔI329L. The reduced protection observed following immunisation with a deletion mutant from an already attenuated virus is not without precedent [16]. Gallardo et al. showed that the deletion of another IFN inhibitor (A276R) from the NH/P68 isolate results in the complete loss of protection against challenge with Arm07, in contrast to the 100% protection afforded by vaccination with the wild-type virus. In our study, the reduced protection in pigs immunised with OURT88/3ΔI329L was associated with an impairment of both antibody and cellular immune responses, as measured by VP72 ELISA and IFN-*γ* ELIspot, respectively. The reasons for this are unknown and may involve reduced virus replication in the non-protected pigs, altered immune responses or both. Interestingly, type I IFN has been implicated in the dysregulation of the immune responses during persistent viral infections [17,18]. Similarly, during ASFV infection, a delicate control of the IFN response may be necessary to avoid viral persistence and to promote the induction of protective responses. Hence, enough IFN needs to be produced to control early virus replication and to stimulate adaptive immune responses but excessive/prolonged IFN exposure may result in immunosuppression. Whilst we did not observe an increase in IFN-*α* levels in the serum of non-protected animals following vaccination, we cannot dismiss the notion that high levels of IFN were present at the viral replication sites. Comparably to Golding et al., [19] we observed a sharp increase in type I IFN levels following the challenge of control pigs with OURT88/1, which is probably associated with high levels of viral replication and does not correlate with the ability of the virus to control the IFN response in vitro.

Levels of IL-10 were significantly higher at day 3 post-challenge in the group immunised with OURT88/3ΔI329L, and this was mainly driven by those animals that did not survive challenge. IL-10 prevents excessive inflammatory responses and is a broadly expressed cytokine, known to be produced by cells of the innate immune system (macrophages, dendritic cells and natural killer cells among others) and cells of the adaptive immune system including regulatory T (Tregs) cells but also T helper (Th) cells, cytotoxic T lymphocytes (CTLs) and B cells [20]. In the current study, infected macrophages are unlikely to be the source of the high levels of IL-10 observed in the vaccinated/non-protected pigs, since IL-10 values were much lower in control pigs, which presented the highest viremias at this day post-challenge. Furthermore, a recent study [21] showed that IL-10 was expressed at significantly lower levels in cells infected with the highly virulent Georgia/2007 isolate than in non-infected cells. Taken together, these results indicate that, in the group immunised with OURT88/3Δ329L, IL-10 is probably produced by cells of the adaptive immune system. It is therefore tempting to speculate that immunisation with the deletion mutant virus resulted in an increase in the number of local and/or circulating Tregs. Two main subsets of Tregs have been described: naturally occurring Tregs (nTregs) and inducible Tregs (iTregs). iTregs may be classified as T_R_1 (IL-10 producing) or T_H_3 (TGF-*β* producing) [22] and they can be induced by prolonged exposure to circulating antigen, chronic inflammation or weak co-stimulation in the periphery [23]. The deletion of the I329L gene, an inhibitor of TLR signalling [7] might have indeed increased the inflammatory response at the viral replication sites, thus promoting Treg induction. Interestingly, a recent study found a correlation between the lack of protection following the immunisation of pigs with OURT88/3 and increased levels of IL-10 and Tregs [24]. It is also interesting to note that type I IFN signalling has been implicated in the promotion of T_R_1 responses during chronic virus infection [25], and, therefore, the relationship between IFN responses, the activation of regulatory T cells and IL-10 production in the context of ASFV infection merits further studies.

The single deletion of the I329L gene did not attenuate the virulent Georgia/2007 isolate. This is probably a result of its functional redundancy, since several other IFN inhibitors are encoded by ASFV. As discussed above, the attenuation of this isolate required the deletion of multiple members of the MGF 360 and 505 [3]. Additionally, I329L was shown to inhibit the IFN induction pathway when activated via TLR signalling but not through the engagement of intracellular receptors [7]. Our results indicate that attenuation may require the deletion of gene(s) acting downstream in the signalling cascade (e.g., at the level of transcription factors) that allow the virus to control IFN expression induced by different receptors, including the cytoplasmic DNA sensor cyclic guanosine monophosphate–adenosine monophosphate (GMP-AMP) synthase (cGAS). It was also shown [26] that the Armenia/07 isolate, a virus closely related to Georgia/2007, is able to control IFN induction through the cGAS-STING pathway. Therefore, the identification of ASFV genes involved in the modulation of host DNA sensing may provide additional targets for the development of rationally attenuated ASFV vaccines.
